# Accidental Durotomy in Minimally Invasive Transforaminal Lumbar Interbody Fusion: Frequency, Risk Factors, and Management

**DOI:** 10.1155/2015/532628

**Published:** 2015-05-17

**Authors:** Jan-Helge Klingler, Florian Volz, Marie T. Krüger, Evangelos Kogias, Roland Rölz, Christoph Scholz, Ronen Sircar, Ulrich Hubbe

**Affiliations:** Department of Neurosurgery, Freiburg University Medical Center, 79106 Freiburg, Germany

## Abstract

*Purpose*. To assess the frequency, risk factors, and management of accidental durotomy in minimally invasive transforaminal lumbar interbody fusion (MIS TLIF). *Methods*. This single-center study retrospectively investigates 372 patients who underwent MIS TLIF and were mobilized within 24 hours after surgery. The frequency of accidental durotomies, intraoperative closure technique, body mass index, and history of previous surgery was recorded. *Results*. We identified 32 accidental durotomies in 514 MIS TLIF levels (6.2%). Analysis showed a statistically significant relation of accidental durotomies to overweight patients (body mass index ≥25 kg/m^2^; *P* = 0.0493). Patient age older than 65 years tended to be a positive predictor for accidental durotomies (*P* = 0.0657). Mobilizing patients on the first postoperative day, we observed no durotomy-associated complications. *Conclusions*. The frequency of accidental durotomies in MIS TLIF is low, with overweight being a risk factor for accidental durotomies. The minimally invasive approach seems to minimize durotomy-associated complications (CSF leakage, pseudomeningocele) because of the limited dead space in the soft tissue. Patients with accidental durotomy can usually be mobilized within 24 hours after MIS TLIF without increased risk. The minimally invasive TLIF technique might thus be beneficial in the prevention of postoperative immobilization-associated complications such as venous thromboembolism. This trial is registered with DRKS00006135.

## 1. Introduction

Surgical fusion techniques are used to treat degenerative, infectious, and traumatic pathologies of the lumbar spine [1–3]. The traditional open technique includes a long midline skin incision with dissection and retraction of the paravertebral musculature to expose the posterior structures of the spine. Minimally invasive techniques were shown to be equally effective as open procedures while leading to reduced blood loss, less postoperative morbidity, and faster recovery [4–6]. Accidental durotomies are an undesirable intraoperative complication with a reported frequency of 3.2 to 18.5% in spine surgery [7–12]. In minimally invasive transforaminal lumbar interbody fusion (MIS TLIF), however, accidental durotomies have scarcely been investigated [7].

We hypothesized that the frequency of accidental durotomies in MIS TLIF is comparable to other lumbar procedures.

## 2. Methods

### 2.1. Ethics Statement

The local ethics committee approved the study. The study is registered in the German Clinical Trials Register (DRKS00006135).

### 2.2. Data Collection

Through a retrospective review of our institutional database, we identified 372 consecutive patients (218 women and 154 men, age: 64.6 ± 13.6 years) who underwent MIS TLIF in 514 levels between January 2006 and March 2014. The vast majority of patients had degenerative disease (98.4%); few patients were operated on due to infection (1.6%). Based on operation reports and medical records, we collected details on the demographics, level of surgery, history of previous surgery at the same level, extent of decompression, intraoperative technique of closing durotomies, and durotomy-related complications. The frequency of accidental durotomies and their potential association with the body mass index (BMI), patient age, and history of previous surgery at the same level were investigated.

### 2.3. Surgical Technique

**Figure 1:**
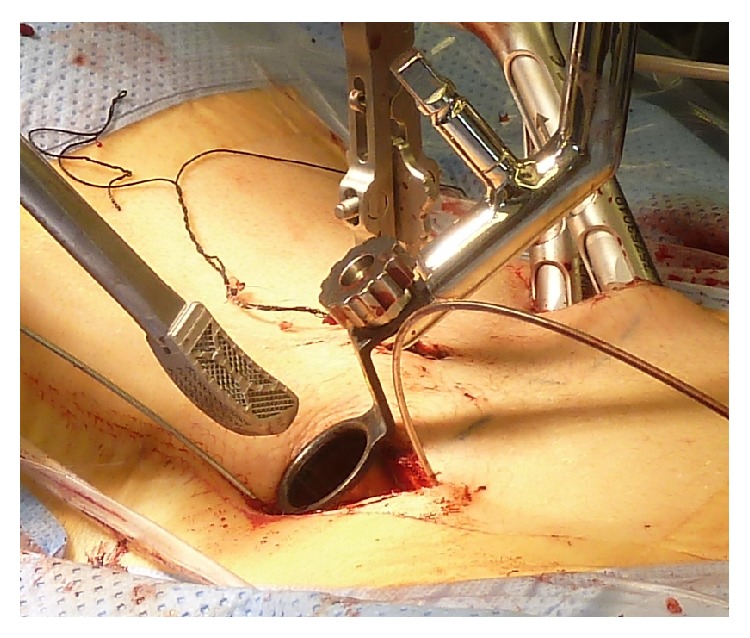
Minimally invasive cage implantation. An intervertebral cage made of titanium mesh is introduced minimally invasively through a nonexpendable tubular retractor (20 mm diameter) into the intervertebral disc space.

**Figure 2:**
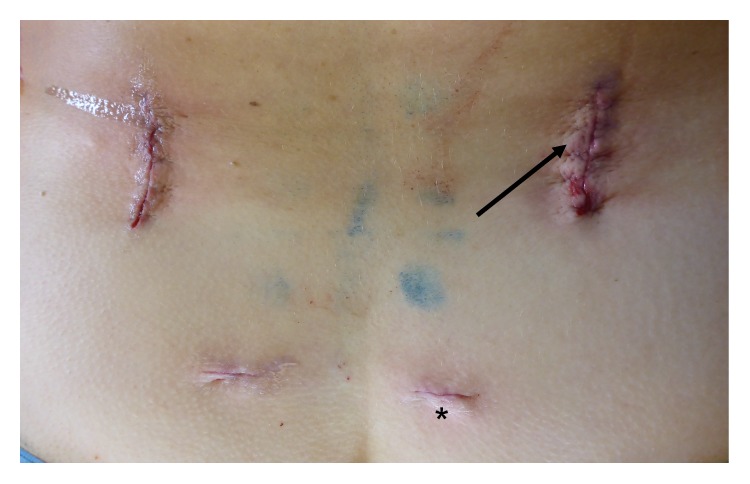
Wound closure. Subcutaneous sutures and skin adhesive were used for closing the wound. Placement of a drain is not necessary. The more lateral incisions (arrows) were used for minimally invasive implantation of screws, decompression of the spinal canal, and transforaminal insertion of the intervertebral cage (see also Figure 1). The stab incisions (asterisk) were used for minimally invasive rod insertion.

Each patient was placed in prone position on a radiolucent table under general anesthesia. Via bilateral short skin incisions (3 cm), a Jamshidi needle was introduced into the target vertebras through the pedicles using 3D C-arm navigation or C-arm fluoroscopic images, and Kirschner wires were inserted through the Jamshidi needle. A minimally invasive, transmuscular approach was created using a nonexpandable tubular retractor system (METRx, Medtronic, Minneapolis, USA). An operating microscope was used during the following transforaminal access including facetectomy, unilateral or bilateral decompression of the spinal canal and partial discectomy. After implantation of a TLIF cage (Figure 1), the 360-degree fusion was completed by insertion of cannulated screws via the Kirschner wires and minimally invasive rod insertion (e.g., CD Horizon Sextant II, CD Horizon Sextant Solera, and CD Horizon Longitude (Medtronic, Minneapolis, USA)). If bone density was considered to be low, additional cement augmentation of vertebral bodies was performed under fluoroscopic image guidance. Skin was closed with subcutaneous sutures and skin adhesive without placing a drain (Figure 2).

**Figure 3:**
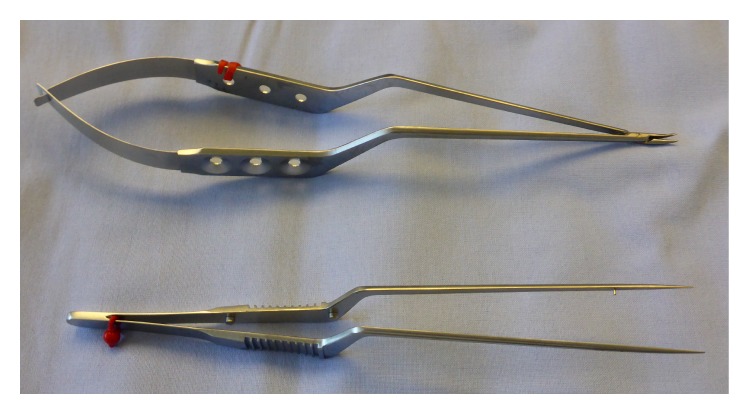
Microsurgical instruments. The figure shows a bayonet microneedle holder and bayonet microforceps that were used for suturing accidental durotomies.

In case of durotomy, the dura was closed with nonresorbable suture (5/0 PremiCron, B. Braun, Melsungen, Germany) where possible and supported by a fibrinogen/thrombin-coated sponge (TachoSil, Takeda, Berlin, Germany) or gelfoam with fibrin glue to the surgeon's discretion. Commercially available, microsurgical instruments (bayonet microneedle holder and bayonet microforceps) were used for suturing the durotomy (Figure 3).

All patients, independent of occurrence of accidental durotomies, were mobilized within 24 hours of surgery unless the patient's clinical status prohibited mobilization.

### 2.4. Statistical Analysis

Results were expressed as mean with standard deviation. Statistical comparisons for categorical values between groups were accomplished using the two-tailed Fisher exact test. Prism 6 for Mac (GraphPad Software Inc., La Jolla, USA) and Excel 2011 for Mac (Microsoft Corporation, Redmond, USA) were used as statistical software and for data processing. *P* values < 0.05 were considered to be statistically significant.

## 3. Results

### 3.1. Frequency of Accidental Durotomies

Thirty-two accidental durotomies occurred in 514 MIS TLIF levels (6.2%) (Table 1).  Table 2 shows the distribution of operated levels and accidental durotomies. MIS TLIF was performed most frequently at L4/5 (41.8%); furthermore, the highest percentage of durotomies was registered at L4/5 (9.8%).

### 3.2. Durotomy and Body Mass Index, Age, or Previous Surgery

We investigated a possible association between accidental durotomies and the BMI, patient age, and history of previous surgery at the same level.

Overweight patients (BMI ≥ 25 kg/m^2^) had a higher incidence of durotomies (7.8% versus 3.0%, Table 3). The two-tailed Fisher exact test showed a statistically significant relation of accidental durotomies to overweight patients (*P* = 0.0493). Patients with an age of at least 65 years had a higher incidence of durotomies (7.9% versus 3.8%, Table 4). The two-tailed Fisher exact test showed no statistically significant relation between accidental durotomies and age cohort (*P* = 0.0657). Patients with history of previous surgery in the level of MIS TLIF had a higher incidence of durotomies (8.3% versus 5.3%, Table 5). The two-tailed Fisher exact test showed no statistically significant relation between accidental durotomies and history of previous surgery in the level of MIS TLIF (*P* = 0.1535).

### 3.3. Adhesions and Scarring

In 11 of the 32 levels with accidental durotomy (45.0%), neither history of previous surgery nor adhesions were recorded (Table 1). Twelve of the 32 levels (37.5%) with accidental durotomies had a history of previous decompression surgery. Six of these (50.0%) were stated in context with scar tissue that was adherent to the lacerated dura. Adhesions with subsequent accidental durotomy were found in 9 of 20 levels without history of previous surgery. Altogether, adhesions or scarring was found in 15 of 32 levels (46.9%), independent of a history of previous surgery.

### 3.4. Postoperative Complications

None of the patients with accidental durotomies developed a postoperative CSF fistula or needed revision surgery due to durotomy-associated complications. Apart from durotomy-associated complications, three patients experienced postoperative complications. Patient number 10 sustained an accidental durotomy during cage implantation with concomitant direct nerve injury. Postoperatively, the patient had new flexion and extension paresis of the right foot. Patient number 17 postoperatively sustained a transitory psychotic syndrome. After recovering from this syndrome, a hindered mobilization with an increased local pain level could be recognized. Imaging revealed a postoperative epidural hemorrhage, which was surgically evacuated without clinical sequelae. Patient number 28 sustained multiple organ failure and died on postoperative day 8.

## 4. Discussion

Accidental durotomies can lead to persistent CSF leakage with formation of a pseudomeningocele and CSF leak syndrome. The resultant symptoms include postural headache, nausea, back pain, intracranial hemorrhage, neurological deficits, and meningitis [8, 13]. Thus, if an accidental durotomy occurs, the surgeon has to ensure proper dural closure intraoperatively to prevent persistent CSF leakage. But even if accidental durotomy does occur in minimally invasive spine surgery, it is thought to be much less likely to cause sequelae because there is barely dead space available for formation of a pseudomeningocele. The underlying cause is that the paraspinal musculature is not dissected during the approach and slides back to its original position after the tubular retractor has been removed [9, 10, 14].

### 4.1. Frequency of Accidental Durotomies

The present literature regarding the incidence and management of accidental durotomies in MIS TLIF is very limited and includes only smaller patient cohorts [7, 10, 15]. Senker et al. [7] retrospectively identified 10 accidental durotomies in 72 patients (13.9%) who underwent MIS TLIF or percutaneous lumbar stabilization. Half of the durotomies were closed with sutures. Sulaiman and Singh [15] reported 1 durotomy in 57 patients with MIS TLIF (1.8%) and 1 durotomy in 11 patients with open TLIF (9.1%). Than et al. [10] observed durotomies in 6.3% of 112 patients with minimally invasive lumbar spine procedures (decompressive and fusion procedures). Telfeian et al. [9] observed accidental durotomies in 16.7% of 12 patients with severe obesity (BMI > 40 kg/m^2^) who underwent heterogeneous spine operations. Ruban and O'Toole [8] retrospectively examined minimally invasive operations (decompressive and fusion procedures) of the whole spine and found durotomies in 9.4% of 563 patients. Ross [16] stated an incidence for lumbar durotomies in 3.4% of 929 cases during minimally invasive decompression surgery. Khan et al. [17] reported an incidence for lumbar durotomies in 10.6% of 3,183 patients (decompressive and fusion procedures); the subgroup with history of previous surgery at the same level showed a higher incidence of 15.9%. Studies comparing open TLIF and open PLIF (posterior lumbar interbody fusion) procedures found higher numbers of durotomies in open PLIF procedures (17.1% (13/76 patients) versus 9.3% (4/43 patients) [18]; 7.7% (4/52 patients) versus 0.0% (0/50 patients) [19]).

Thus, the rate of accidental durotomies in our study with 6.2% in 514 levels with MIS TLIF is comparably low. Only two other studies with considerably less numbers of patients investigated durotomies in MIS TLIF procedures as well and reported durotomies in 1.8% [15] and 13.9% [7] of them, respectively.

### 4.2. Risk Factors

Accidental durotomies have been reported to occur more often in patients with older age, scars from previous surgery, ossificated ligamentum flavum, thinning of dura attributable to chronic compression, and surgeon inexperience [8, 10, 17, 20, 21]. In general, revision surgery is more demanding than primary surgery due to scarring and modifications of the anatomy. Thus, revision surgery may be associated with higher complication rates such as accidental durotomies or nerve root injury [8, 12, 17]. Likewise, levels with history of previous surgery showed an increased durotomy rate in our study (8.8% versus 5.3%, Table 5), though without reaching statistical significance. Our study demonstrated that accidental durotomies occur more often in overweight patients compared to normal weight patients. The longer approach to the spinal canal and, thus, the more difficult dissection with longer instruments might be an explanation for this finding. Consistent with Senker et al. [7], we did not find a statistical difference regarding durotomies between patients who were younger or older than 65 years (*P* = 0.0657), although the older age cohort tended to sustain more durotomies (7.9% versus 3.8%, Table 4).

### 4.3. Use of Drain

We generally use no drain in minimally invasive surgery. We agree with other authors [8, 10] that particularly the minimally invasive approach with its rather small corridor to the spine allows the soft tissue to slip back after removal of the tubular retractor and thus counters CSF accumulation in case of accidental durotomy. Thus, placing a drain is not necessary in our opinion. Moreover, we do not believe that placement of a subfascial drain prevents hematomas as stated by other authors [7]. Nevertheless, the use of drains after accidental durotomy in minimally invasive spine surgery remains controversial, since some authors describe their routine use [17, 22].

### 4.4. Mobilization after Durotomy

Early mobilization is recommended in elective spine surgery to reduce postoperative complications like venous thromboembolism [23]. We aimed to mobilize all patients on the first postoperative day, independent of occurrence of durotomy. Twenty-one of 30 patients with accidental durotomy could be mobilized on the first postoperative day (Table 1) without any complication. We therefore agree with Ruban and O'Toole [8] who also mobilized patients with durotomies within 24 hours of minimally invasive surgery without any complications. Than et al. [10] mobilized patients with durotomy within 48 hours after minimally invasive surgery without any complication, and Senker et al. [7] applied bed rest for 2.5 to 5 days in case of accidental durotomies after minimally invasive procedures. In contrast, bed rest after a durotomy during open spinal procedures has been recommended for up to 7 days [8, 17, 22], supporting that minimally invasive approaches are beneficial for early mobilization, even after durotomy. The underlying theory is that the minimally invasive approach with small skin incisions and the muscle-dilating technique causes only a very limited dead space in the soft tissue. This decreased space is believed to create less potential for CSF accumulation, permanent CSF leakage, and formation of a pseudomeningocele in comparison to the open approach [8, 10]. In this regard, we consider MIS TLIF superior to open TLIF or PLIF as patients with durotomy do not require extended bed rest after MIS TLIF and are recommended to be mobilized within 24 hours of surgery. Early mobilization is preventive regarding postoperative complications [23] and potentially reduces the length of hospital stay.

## 5. Conclusions

The frequency of accidental durotomies in MIS TLIF is low, and overweight is a risk factor for accidental durotomies. The minimally invasive approach seems to minimize durotomy-associated complications such as permanent CSF leakage or pseudomeningocele because of the limited dead space in the soft tissue. Furthermore, patients with accidental durotomy can usually be mobilized within 24 hours after MIS TLIF without increased risk of complications. The minimally invasive TLIF technique might thus be beneficial in the prevention of postoperative immobilization-associated complications such as venous thromboembolism.

## Limitations of the Study

The retrospective design is an obvious methodological weakness of this trial. Since patients were retrospectively included, no power analysis was performed. Furthermore the study lacks an open TLIF control group and does not compare differing durotomy repair strategies.

## Figures and Tables

**Table 1:** Characterization and dural closure technique in patients with accidental durotomies.

Patient number	Sex	Age (years)	BMI (kg/m^2^)	Level of durotomy	Contralateral decompression?	Site of durotomy	Previous surgery?	Adhesions scarring?	Durotomy during	Closure	Mobilization
1	Male	76.4	29.8	L4/5	Yes	Ipsilateral	Yes	No	Cage implantation	Suture + TachoSil	1st postop. day

2	Female	73.0	27.0	L3/4	Yes	Ipsilateral	No	Yes	Cage implantation	TachoSil	3rd postop. day
				L4/5	Yes	Contralateral	No	Yes	Decompression	TachoSil	

3	Female	79.6	27.8	L3/4	Yes	Ipsilateral	No	Yes	Decompression	TachoSil	3rd postop. day
				L4/5	Yes	Contralateral	No	Yes	Decompression	TachoSil	

4	Male	68.6	38.2	L2/3	Yes	Ipsilateral	Yes	Yes	Decompression	TachoSil	1st postop. day

5	Male	71.6	33.2	L3/4	No	Ipsilateral	Yes	No	Cage implantation	TachoSil	1st postop. day

6	Female	65.2	32.3	L4/5	Yes	Contralateral	No	Yes	Decompression	TachoSil	1st postop. day

7	Female	71.4	34.0	L4/5	Yes	Ipsilateral	Yes	Yes	Decompression	TachoSil	3rd postop. day

8	Female	89.7	26.7	L4/5	Yes	Contralateral	No	Yes	Decompression	TachoSil	1st postop. day

9	Male	65.6	24.6	L4/5	Yes	Contralateral	No	No	Decompression	TachoSil	1st postop. day

10	Female	55.7	34.1	L3/4	Yes	Ipsilateral	No	No	Cage implantation	Suture + TachoSil	1st postop. day

11	Male	58.2	25.3	L5/S1	No	Ipsilateral	No	No	N/A	TachoSil	1st postop. day

12	Female	59.6	27.1	L4/5	Yes	N/A	No	No	Decompression	Suture + TachoSil	1st postop. day

13	Female	78.9	22.1	L3/4	Yes	Ipsilateral	Yes	No	Cage implantation	Gelfoam + fibrin glue	2nd postop. day

14	Male	66.2	28.4	L4/5	No	Ipsilateral	No	No	Decompression	TachoSil	1st postop. day

15	Male	64.4	34.3	L4/5	No	Ipsilateral	Yes	No	Decompression	TachoSil	1st postop. day

16	Female	55.0	33.8	L4/5	No	Ipsilateral	Yes	Yes	Decompression	TachoSil	1st postop. day

17	Female	74.2	44.3	L3/4	Yes	Ipsilateral	No	No	Decompression	Suture + TachoSil	6th postop. day due to transitory psychotic syndrome

18	Male	73.6	27.6	L4/5	No	Ipsilateral	Yes	Yes	Decompression	Suture + TachoSil	1st postop. day

19	Male	62.4	27.8	L4/5	Yes	Ipsilateral	No	Yes	Decompression	Suture + TachoSil	1st postop. day

20	Female	78.8	34.9	L2/3	No	Ipsilateral	Yes	No	Decompression	TachoSil	2nd postop. day

21	Female	71.7	27.3	L3/4	No	Ipsilateral	Yes	No	Decompression	Suture + TachoSil	1st postop. day

22	Female	75.3	31.0	L4/5	Yes	Ipsilateral	No	Yes	Decompression	Suture + TachoSil	1st postop. day

23	Male	66.0	24.8	L4/5	Yes	Ipsilateral	No	No	Decompression	TachoSil	1st postop. day

24	Male	62.5	30.7	L4/5	Yes	Ipsilateral	Yes	Yes	Cage implantation	TachoSil	1st postop. day

25	Female	75.2	27.9	L4/5	Yes	Contralateral	No	No	Decompression	TachoSil	5th postop. day

26	Female	70.1	40.4	L5/S1	Yes	Contralateral	No	No	Decompression	TachoSil	2nd postop. day

27	Female	65.5	24.2	L4/5	No	Ipsilateral	No	No	Decompression	TachoSil	1st postop. day

28	Male	78.5	24.4	L4/5	No	Ipsilateral	No	No	Decompression	Suture	N/A (died of multiple organ failure on postop. day 8)

29	Male	79.4	26.1	L4/5	Yes	Ipsilateral	No	Yes	Decompression	Suture	1st postop. day

30	Male	44.4	29.2	L4/5	No	Ipsilateral	Yes	Yes	Decompression	None (due to intact arachnoidea)	1st postop. day

Mean		69.2	30.0								1.6

Standard deviation		9.3	5.1								1.3

	16 × female			2 × L2/3	21 × yes	24 × ipsilateral	12 × yes	15 × yes	25 × decompression	20 × TachoSil	

	14 × male			7 × L3/4	11 × no	7 × contralateral	20 × no	17 × no	6 × cage implantation	8 × Suture + TachoSil	

				21 × L4/5		1 × N/A			1 × N/A	2 × suture	

				2 × L5/S1						1 × gelfoam + fibrin glue	

										1 × none	

BMI: body mass index.

N/A: not available.

**Table 2:** Distribution of operated levels and accidental durotomies.

Level of MIS TLIF	Number of MIS TLIF	Number of durotomies	Percentage of durotomies
L1/2	14	0	0.0%
L2/3	49	2	4.1%
L3/4	96	7	7.3%
L4/5	215	21	9.8%
L5/S1	140	2	1.4%

Total	514	32	6.2%

MIS TLIF: minimally invasive transforaminal lumbar interbody fusion.

**Table 3:** Number and relation of accidental durotomies and BMI.

Durotomy		BMI (kg/m^2^)		Total
		<25	≥25	
	**Levels operated**	**162**	**320**	**482**

No	% without durotomy	33.6%	66.4%	100%
	% within BMI group	97.0%	92.2%	

	**Levels operated**	**5**	**27**	**32**

Yes	% with durotomy	15.6%	84.4%	100%
	% within BMI group	3.0%	7.8%	

The two-tailed Fisher exact test showed a statistically significant relation of accidental durotomies to overweight patients (*P* = 0.0493).

BMI: body mass index.

**Table 4:** Number and relation of accidental durotomies and patient age.

Durotomy		Age		Total
		<65 years	≥65 years	
	**Levels operated**	**201**	**281**	**482**

No	% without durotomy	41.7%	58.3%	100%
	% within age cohort	96.2%	92.1%	

	**Levels operated**	**8**	**24**	**32**

Yes	% with durotomy	25.0%	75.0%	100%
	% within age cohort	3.8%	7.9%	

The two-tailed Fisher exact test showed no statistically significant relation between accidental durotomies and age cohort (*P* = 0.0657).

**Table 5:** Number and relation of accidental durotomies and history of previous surgery.

Durotomy		Previous surgery		Total
		No	Yes	
	**Levels operated**	**357**	**125**	**482**

No	% without durotomy	74.1%	25.9%	100%
	% within group of previous surgery	94.7%	91.7%	

	**Levels operated**	**20**	**12**	**32**

Yes	% with durotomy	62.5%	37.5%	100%
	% within group of previous surgery	5.3%	8.3%	

The two-tailed Fisher exact test showed no statistically significant relation between accidental durotomies and history of previous surgery in the level of MIS TLIF (*P* = 0.1535).

## Abbreviations

BMI: Body mass index
CSF: Cerebrospinal fluid
MIS TLIF: Minimally invasive transforaminal lumbar interbody fusion
PLIF: Posterior lumbar interbody fusion.
